# The clinical impact of hepatitis C virus infection in Egyptian multiple myeloma patients

**DOI:** 10.1186/s43046-020-00054-0

**Published:** 2020-11-27

**Authors:** Neemat M. Kassem, Hebatallah A. Kassem, Magdy Ibrahim, Hussam Zawam, Emad Hamada

**Affiliations:** 1 Clinical and Chemical Pathology Department, Kasr Al Ainy Centre of Clinical Oncology & Nuclear Medicine, School of Medicine, Cairo University, Cairo, Egypt; 2 Gynecology & Obstetric Department, School of Medicine, Cairo University, Cairo, Egypt; 3 Clinical Oncology Department, School of Medicine, Cairo University, Cairo, Egypt

**Keywords:** Hepatitis C virus, Multiple myeloma, Prevalence, Hepatic adverse events

## Abstract

**Background:**

Multiple myeloma (MM) is a human B cell neoplasia characterized by the clonal proliferation of malignant plasma cells in the bone marrow. Worldwide, hepatitis C virus (HCV) infection is a public health problem. For MM patients, the clinical impact of preexisting HCV infection is still unclear. We aim to assess the clinical characteristics and the prevalence of the HCV infection in Egyptian MM patients. This observational study included 81 MM patients. HCV antibody assay was performed, and positive cases were confirmed using a reverse transcription-quantitative PCR (RT-PCR) method.

**Results:**

Fifteen (18.5%) patients were anti-HCV antibody positive. Only 6/15 (7.4%) patients were HCV RNA positive by RT-PCR. Liver affection in the form of hyperbilirubinemia with grade 4 adverse events was significantly higher in the anti-HCV positive/HCV RNA positive group versus anti HCV negative group (16.7% vs. 1.5%, *p* value = 0.005). The median HCV-RNA before the initiation of chemotherapy was 2.5 log IU/ml with mean ± SD = 4.25 ± 1.6 with no HCV reactivation. In the univariate and multivariate analysis, HCV infection was not an independent factor related to DFS. Low hemoglobin level < 10 g/dL (HR 0.59, 95% CI, 0.36–0.97, *p* value = 0.037) and abnormal serum total bilirubin level (HR 1.9, 95% CI 1.03–3.5, *p* value = 0.039) influenced DFS in the univariate analysis. However, in the multivariate analysis, serum calcium level greater than 12 mg/dL (HR 7.04, 95% CI 1.12–44.45, *p* value = 0.038) and abnormal serum total bilirubin level (HR 10.9, 95% CI 2.92–41.02, *p* value = < 0.001) remained statistically significant worse prognostic factors.

**Conclusion:**

In conclusion, our study revealed the prevalence of HCV infection in Egyptian MM patients. Serologic tests at diagnosis are necessary to identify these patients, and confirmation of positive cases by molecular techniques should be mandatory, with regular follow-up for liver dysfunction. Finally, further larger studies explaining the molecular mechanisms linking HCV and the MM pathogenesis are warranted.

## Background

Multiple myeloma (MM) is a human B cell neoplasia characterized by the clonal proliferation of malignant plasma cells (PCs) in the bone marrow (BM) [1]. MM has been raised from an asymptomatic premalignant proliferation of monoclonal plasma cells that are derived from post-germinal-center B cells. The etiology of MM is poorly understood. Genetic and micro-environmental changes induce transformation of these premalignant cells into the malignant stage. Genomic studies suggest that a dominant mutation cluster has been identified in *RAS/RAF* genes, emphasizing the potential significance of RAS/RAF/MEK/ERK signaling as a therapeutic target [2]. Mutation of genes such as i*KRAS*, *NRAS*, *TP53*, *FAM46C*, *DIS3*, and *BRAF* has a high recurrence rate and may play important roles in the pathogenesis, progression, and prognosis of MM [3]. Polymerase chain reactions (PCR), stripassay, and sequencing studies have been used to determine such genetic mutations in cancer patients [4]. Also, interactions between myeloma cells and BM stromal cells or extracellular matrix proteins that are mediated through cell-surface receptors (e.g., integrins, cadherins, selectins, and cell-adhesion molecules) increase tumor growth, survival, migration, and also drug resistance [5]. Despite the recent therapeutic options to treat MM, it remains an incurable condition for the majority of patients, with a median survival of 3–5 years [6]. In Egypt, an estimated 871 new MM cases in 2018 were found, representing 0.68% of all new cancer cases. The number of MM-related deaths accounts for nearly 783 deaths (0.92% of all deaths) [7]. Worldwide, hepatitis C virus (HCV) infection is a public health problem where its seroprevalence had an estimated 2.8% elevation in the last decade, equal to more than 185 million infections (3% of the world’s population) [8]. Recent assessment revealed that 119 million global adult inhabitants have chronic HCV infection, with 3–4 million new infections and 350,000 to 500,000 deaths occurring each year as a result of HCV-related complications. HCV is the main source of chronic hepatitis, liver cirrhosis, and hepatocellular carcinoma (HCC). About 55–85% of HCV-infected patients become chronic active cases passing to fibrosis, cirrhosis, and may progress till decompensated cirrhosis and HCC [9]. In Egypt, HCV infection has been a public health problem. The predominance of HCV infection is highest in Egypt ranging from 6 to 40% with an average of 14%. Approximately, 75% of the individuals are constantly asymptomatic and remain undiagnosed [10]. The association between chronic HCV infection and lymphoproliferative diseases such as non-Hodgkin’s lymphoma, Hodgkin’s lymphoma, and MM has been discussed for a long time [11, 12]. Patients with viral hepatitis infections have been suggested to have a higher risk of MM. However, the association between HCV infection and MM remains controversial [11]. Other various studies revealed a higher incidence of HCV infection in MM patients than healthy control populations [13]. The aim of the current study is to assess the clinical characteristics and the prevalence of the hepatitis C virus infections in Egyptian multiple myeloma patients.

## Methods

### Study population

This observational study included 81 MM patients who were diagnosed as myeloma based on the criteria proposed by the International Myeloma Working Group [6], and all patients were staged at diagnosis according to the International Staging System (ISS). Patients were recruited from our clinical oncology department and were scheduled to receive standard MM treatment throughout the last 2 years. Patients diagnosed with monoclonal gammopathy of undetermined significance (MGUS), POEMS (polyneuropathy, organomegaly, endocrinopathy, monoclonal gammopathy, and skin changes) syndrome, smoldering MM, and solitary plasmacytoma were excluded from this study. Also, cases without HCV serologic test results were excluded. Baseline patient and tumor characteristics, in addition to treatment received were recorded. The study was approved by the Research Ethical Committee of Clinical Oncology department, and informed consents were obtained from all participants prior to enrollment in the study.

### Assessment of HCV infection status

#### Qualitative assessment of hepatitis C virus antibodies

Hepatitis C virus antibody (HCV Ab) assay was performed using the automated ABBOTT Architect i1000 SR system (Singapore, USA). The Architect HCV Ab assay uses chemiluminescent microparticle immunoassay (CMIA) technology for detection of HCV Ab in human serum or plasma blood samples. Negative samples which did not react were considered as non-reactive for HCV antibodies, while reactive samples were considered as positive for HCV antibodies. Reactive samples will be confirmed using a reverse transcription-PCR (RT-PCR) method.

#### Quantitative assessment of hepatitis C virus by RT-PCR

The molecular HCV RNA assay is a confirmatory test with a lower limit of sensitivity of 20 IU/mL which uses RT-PCR technology using StepOne Real-Time PCR machine (Applied Bio-systems, USA). Plasma was isolated from EDTA peripheral blood samples through centrifugation at 800–1600×*g* for 20 min. RNA was extracted from 140 μL of plasma using QIAamp Viral RNA miniKit (Qiagen, Germany) according to the manufacturer’s protocol. The extracted RNA was eluted in elution buffer and used for the quantitative RNA PCR. Quantitative RT-PCR was done according to the manufacturer’s protocol using a commercial HCV kit (Artus HCV RT-PCR CE Kit, Qiagen, Germany). For determination of the viral load, the threshold cycle (Ct) value was used against standard curve to determine the viral load.

#### Hepatitis C virus reactivation

HCV reactivation was defined as tenfold or more increase in the HCV RNA level following chemotherapy compared with the baseline level [14]. Our study compared changes in HCV viral load prior to and after chemotherapy. Acute exacerbation of chronic HCV infection was defined as a threefold or greater elevation in serum ALT level in the absence of (1) infiltration of the liver by tumor, (2) use of hepatotoxic drugs (other than chemotherapeutics), (3) recent blood transfusion (within 1 month of ALT level elevation), or (4) other systemic infections (including hepatitis A, HBV, cytomegalovirus, adenovirus, herpes simplex virus, varicella zoster virus, and human immunodeficiency virus infections) [10].

### Treatment regimen and response to therapy

All MM patients received bortezomib-based therapy that included bortezomib/cyclophosphamide/dexamethasone or bortezomib/thalidomide/dexamethasone for at least 3–4 months in our clinical oncology department. Patients with younger ages (< 60 years old) and high disease burden received induction therapy by VRD (velcade-lenalidomide-dexamethasone). In our study, patients with age > 70 years old to whom the ministry of health does not approve bortezomib therapy, received endoxan-dexamethasone and then maintained by lenalidomide-dexamethasone. Patients with younger age (< 60 years) and no comorbidities who attain complete remission (CR), very good partial response (VGPR), or partial response (PR) were considered for autologous stem cell transplantation (ASCT) then maintenance by lenalidomide-dexamethasone, while those with older age and/or comorbidities were considered for maintenance by lenalidomide-dexamethasone.

### Data analysis

Data were statistically described in terms of mean ± standard deviation (± SD), median and range, or frequencies (number of cases) and percentages when appropriate. Numerical data were tested for the normal assumption using Shapiro-Wilk test. Comparison between HCV status groups was done using Chi-square (χ^2^) test. Exact test was used instead when the expected frequency is less than 5. Survival analysis was done for disease-free survival (DFS) using Kaplan-Meier statistics calculating the mean and median survival time for each group with their 95% confidence interval (CI) and the corresponding survival graphs. Comparison was done between the different factors by Log rank method using Cox-Mantel equation. Univariate and multivariate Cox regression analysis was used to determine the preferential predictors of DFS. Two-sided *p* values less than 0.05 were considered statistically significant. All statistical calculations were done using computer program IBM SPSS (Statistical Package for the Social Science; IBM Corp, Armonk, NY, USA) release 22 for Microsoft Windows.

## Results

**Fig. 1:**
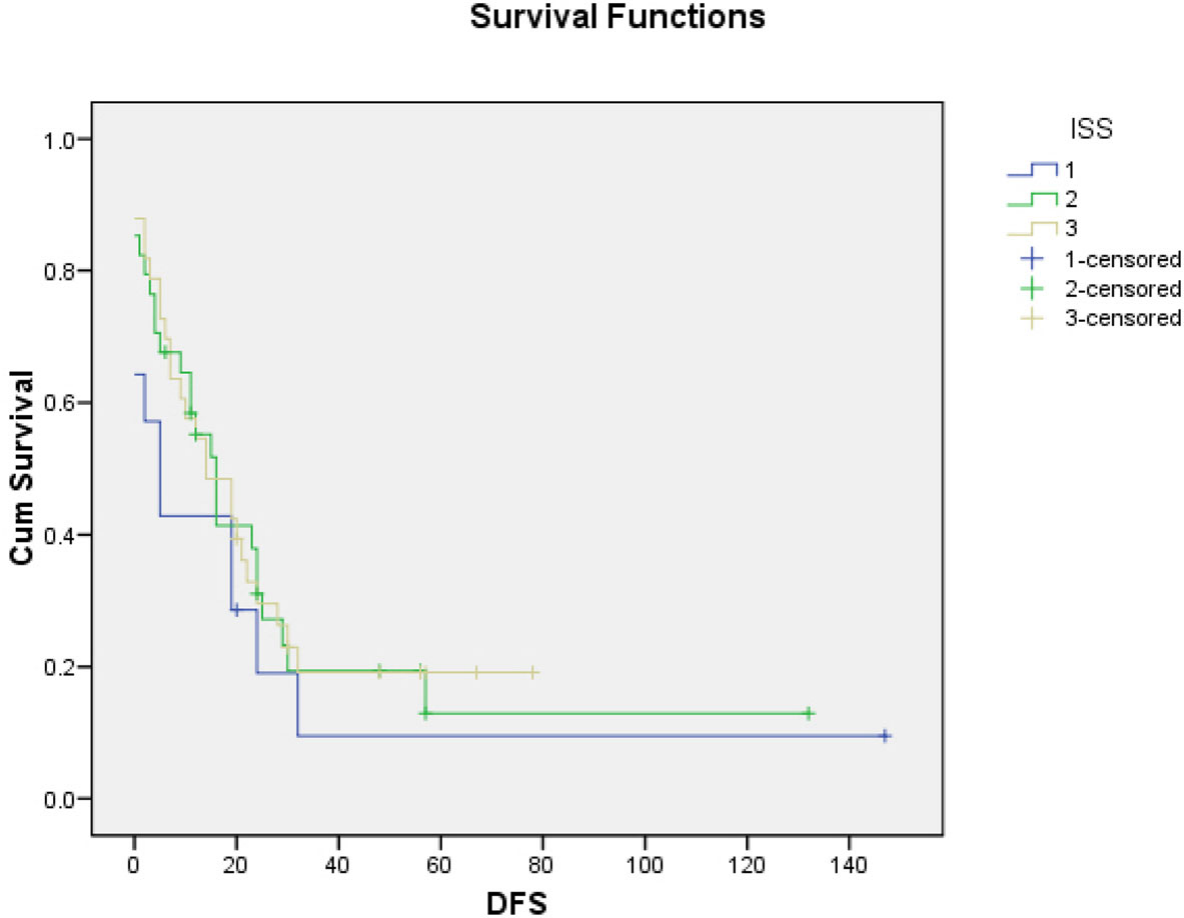
Kaplan-Meier analysis for DFS between the different ISS stages

**Fig. 2:**
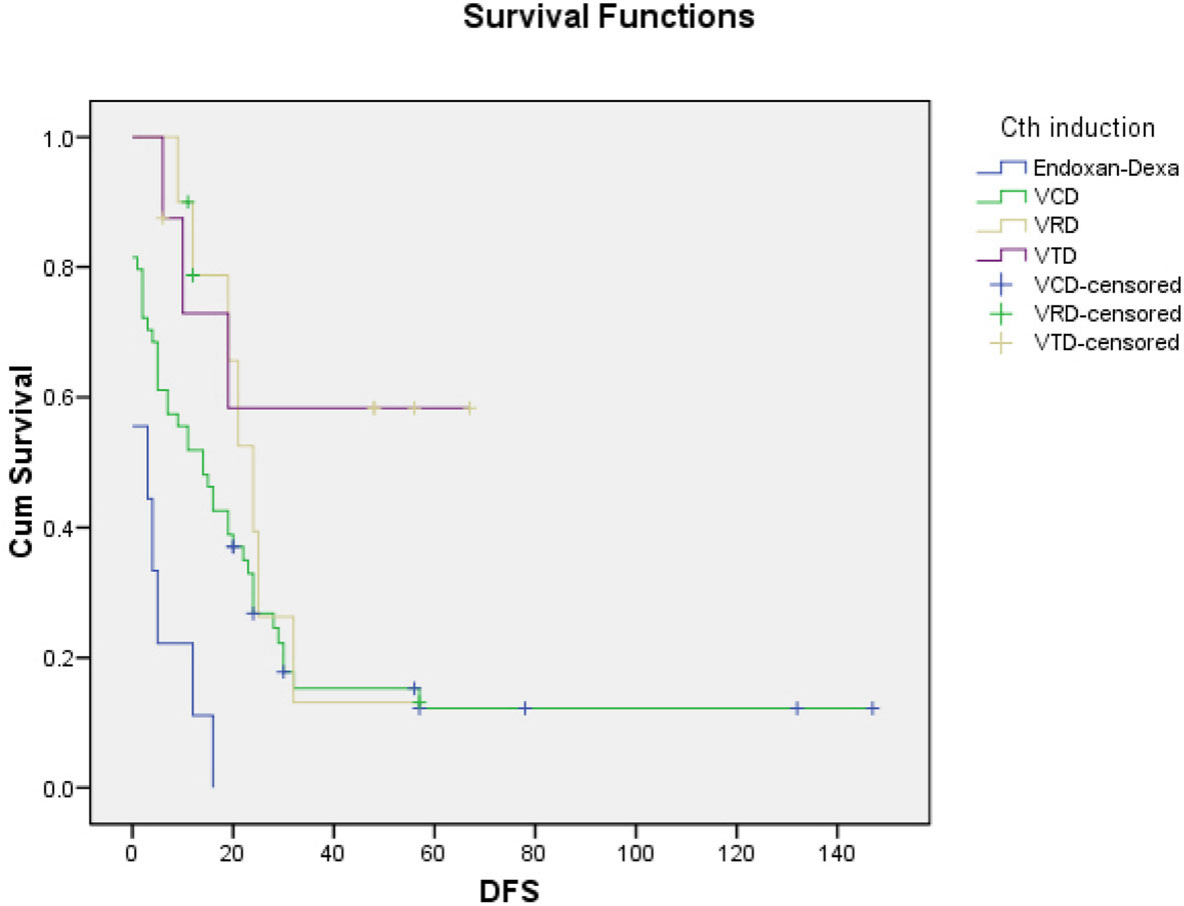
Kaplan-Meier analysis for DFS between the different treatment regimens

Our 81 MM patients were 39 males and 42 females with a male to female ratio 0.93. Their ages ranged between 32 and 75 years with a mean value of 52.2 ± 9.7 years and median of 53 years. According to HCV status, 66/81 (81.5%) patients are anti-HCV negative and 15/81 (18.5%) patients are anti-HCV positive. Out of 15 anti-HCV positive patients, 9 (11.1%) patients are HCV RNA negative by RT-PCR, while the remaining patients (6/15; 7.4%) are HCV RNA positive by RT-PCR. Patients’ baseline clinical and laboratory features are shown in Table 1. To investigate the incidence of hepatic adverse events in multiple myeloma patients, alterations in the ALT, AST, and total bilirubin levels were recorded throughout the treatment course. The incidence of hepatic adverse events during the entire treatment course is shown in Table 2. Regarding ALT and AST elevation, there is no significant difference between anti-HCV negative group and anti-HCV positive group whether HCV RNA by RT-PCR was negative or positive (*p* value = 0.88 and 0.22), respectively. Hyperbilirubinemia with grade 4 adverse events was significantly higher in the anti-HCV positive/HCV RNA positive group versus anti HCV negative group (rates of grade 4 events, 16.7% vs. 1.5%, *p* value = 0.005). The median HCV-RNA before the initiation of chemotherapy was 2.5 log IU/ml with mean ± SD = 4.25 ± 1.6 with no HCV reactivation observed in our study. Three patients received antiviral treatment in the form of sofosbuvir (SOVALDI) by means of the Egyptian national campaign. Antiviral treatments were initiated after the course of MM chemotherapy. These 3 patients achieved sustained viral response (SVR) (HCV-RNA level measured at 3 months after completion of treatments). The other 3 patients died of causes not related to HCV infection, 2 patients received VCD, and their death was attributed to septic shock, severe chest infection, or pneumonia. The other one received VTD, and his death was attributed to intracranial hemorrhage which may be due to marked thrombocytopenia. MM patients were followed up according to available clinical data; the overall survival rate (OS: defined from the date of diagnosis till the date the patient died, or was last seen) and the disease-free survival rate (DFS: defined from the date of CR achievement till the date the patient relapsed) were assessed. OS regarding different MM treatment modalities is described in Table 3. In the univariate and multivariate analysis, HCV infection was not an independent factor related to DFS. Low hemoglobin level < 10 g/dL (HR 0.59, 95% CI 0.36–0.97, *p* value = 0.037) and abnormal serum total bilirubin level (HR 1.9, 95% CI 1.03–3.5, *p* value = 0.039) influenced DFS in the univariate analysis. However, in the multivariate analysis, serum calcium level greater than 12 mg/dL (HR 7.04, 95% CI 1.12–44.45, *p* value = 0.038) and abnormal serum total bilirubin level (HR 10.9, 95% CI 2.92–41.02, *p* value = < 0.001) remained statistically significant worse prognostic factors as shown in Table 4. International staging system (ISS) did not affect DFS (*p* value = 0.587) as described in Table 5 and Fig. 1. Survival analysis according to different MM treatment modalities revealed statistically significant higher DFS rate in patients receiving Velcade® (bortezomib)-thalidomide-dexamethasone as shown in Table 6 and Fig. 2.

**Table 1:** Characteristics of 81MM patients according to their HCV status

Items		No. of patients (%)			***P*** value
		Ab (-)ve No. = 66 (81.5%)	Ab (+)ve/PCR (-)ve No. = 9 (11.1%)	Ab (+)ve/PCR (+)ve No. = 6 (7.4%)	
**Age > 65 years**		3 (4.5%)	2 (22.2%)	0 (0.0%)	0.095
**Gender**	Male	30 (45.5%)	5 (55.6%)	4 (66.7%)	0.545
	Female	36 (54.5%)	4 (44.4%)	2 (33.3%)	
**Serum M-protein:**					
FLC		2 (3.0%)	0 (0.0%)	0 (0.0%)	0.806
IgAK		3 (4.5%)	0 (0.0%)	0 (0.0%)	
IgAL		1 (1.5%)	0 (0.0%)	0 (0.0%)	
IgGK		50 (75.8%)	9 (100.0%)	6 (100.0%)	
IgGL		10 (15.2%)	0 (0.0%)	0 (0.0%)	
**ISS stage:**					
I		14 (21.2%)	0 (0.0%)	0 (0.0%)	0.114
II		24 (36.4%)	7 (77.8%)	3 (50.0%)	
III		28 (42.4%)	2 (22.2%)	3 (50.0%)	
**β2m** ≥ 3.5 mg/L		12 (25.5%)	3 (37.5%)	0 (0.0%)	0.310
**Albumin** < 3.5 g/L		38 (58.5%)	8 (88.9%)	4 (66.7%)	0.205
**Hemoglobin** < 10 g/dL		31 (47.0%)	6 (66.7%)	1 (16.7%)	0.164
**Platelets** < 100 × 10^9^/L		7 (10.6%)	3 (33.3%)	0 (0.0%)	0.096
**Serum calcium** ≥ 12.0 mg/dL		51 (96.2%)	9 (100.0%)	5 (83.3%)	0.762
**Serum creatinine** ≥ 2.0 mg/dL		58 (89.2%)	6 (66.7%)	6 (100.0%)	0.100
**Response to MM treatment:**					
CR		10 (15.2%)	0 (0.0%)	3 (50.0%)	0.100
VGPR		10 (15.2%)	0 (0.0%)	0 (0.0%)	
PR		23 (34.8%)	7 (77.8%)	2 (33.3%)	
PD		13 (19.7%)	1 (11.1%)	0 (0.0%)	
ST		10 (15.2%)	1 (11.1%)	1 (16.7%)	

*HCV* hepatitis C virus, *FLC* free light chain, *β2m* beta-2-microglobulin, *ISS* International Staging System, *ALT* alanine transaminase, *AST* aspartate aminotransferase, *T.bil* total bilirubin, *CR* complete remission, *VGPR* very good partial response, *PR* partial response, *PD* progressive disease, *ST* stationary disease

**Table 2:** Hepatic adverse effects among 81MM patients

Items	No. of patients (%)			***P*** value
	Ab (-)ve *n* = 66 (81.5%)	Ab (+)ve/PCR (-)ve *n* = 9 (11.1%)	Ab (+)ve/PCR (+)ve *n* = 6 (7.4%)	
**Child-Pugh score (patients)**				
A	47 (71.2%)	4 (44.4%)	5 (83.3%)	0.195
B	19 (18.8%)	5 (55.6%)	1 (16.7%)	
**ALT increase**				
Grade 0	65 (98.4%)	9 (100.0%)	6 (100.0%)	0.885
Grade 1	0 (0.0%)	0 (0.0%)	0 (0.0%)	
Grade 2	0 (0.0%)	0 (0.0%)	0 (0.0%)	
Grade 3	1 (1.6%)	0 (0.0%)	0 (0.0%)	
**AST increase**				
Grade 0	57 (86.4%)	6 (66.7%)	3 (50.0%)	0.222
Grade 1	7 (10.6%)	3 (33.3%)	2 (33.3%)	
Grade 2	1 (1.5%)	0 (0.0%)	1 (16.7%)	
Grade 3	1 (1.5%)	0 (0.0%)	0 (0.0%)	
**Total bil. increase**				
Grade 0	56 (85%)	5 (55.6%)	4 (66.7%)	0.005*
Grade 1	3 (4.5%)	4 (44.4%)	1 (16.7%)	
Grade 2	3 (4.5%)	0 (0.0%)	0 (0.0%)	
Grade 3	3 (4.5%)	0 (0.0%)	0 (0.0%)	
Grade 4	1 (1.5%)	0 (0.0%)	1 (16.7%)	

*HCV* hepatitis C virus, *ALT* alanine transaminase, *AST* aspartate aminotransferase, *T.bil* total bilirubin

*Significant at *p* ≤ 0.05

**Table 3:** Overall survival (OS) regarding different treatment regimens

Treatment	Number of patients	OS months		
		Range	(Mean ± SD)	Median
VCD	54	1–156	28.5 ± 30.5	20.3
VRD	10	3–63	27.5 ± 17.7	26.4
VTD	8	12–77	41.5 ± 23.2	44.5
Endoxan-Dexa	9	2–20	8.13 ± 6.9	5.1

*VCD* Velcade® (bortezomib)-cyclophosphamide-dexamethasone, *VRD* Velcade® (bortezomib)-Revlimid® (lenalidomide)-dexamethasone, *VTD* Velcade® (bortezomib)-thalidomide-dexamethasone; *Endoxan-Dexa* endoxan-dexamethasone

**Table 4:** Univariate and multivariate analyses for DFS in MM patients

Items	Univariate			Multivariate		
	HR	95% CI	***P*** value	HR	95% CI	***P*** value
HCV status	0.741	0.462, 1.189	0.214	0.797	0.386, 1.645	0.540
Age (≥ 65 vs. < 65)	1.545	0.553, 4.315	0.407	1.782	0.340, 9.337	0.494
Gender (male vs. female)	1.003	0.613, 1.643	0.989	0.986	0.429, 2.262	0.973
ISS stage	0.865	0.615, 1.214	0.406	1.367	0.558, 3.352	0.494
β2m (≥ 3.5 vs. < 3.5 mg/L)	0.941	0.475, 1.863	0.861	0.329	0.057, 1.89	0.213
Albumin (< 3.5 vs ≥ 3.5 g/L)	1.058	0.639, 1.749	0.827	1.282	0.504, 3.262	0.602
Hemoglobin (< 10 vs. ≥ 10 g/dL)	0.592	0.361, 0.970	0.037*	0.604	0.284, 1.287	0.191
Platelets (< 100 vs. ≥ 100 × 109/L)	0.780	0.385, 1.579	0.490	0.784	0.072, 7.763	0.808
Serum calcium (≥ 12 vs. < 12 mg/dL)	3.253	0.767, 13.795	0.110	7.042	1.12, 44.45	0.038*
Serum creatinine (≥ 2.0 vs. < 2.0 mg/dL	1.123	0.534, 2.362	0.760	0.741	0.162, 3.384	0.699
Serum ALT (abnormal vs. normal)	0.973	0.236, 4.003	0.969	--	--	--
Serum AST (abnormal vs. normal)	1.055	0.545, 2.042	0.874	0.472	0.125, 1.782	0.268
Serum T. bil. (abnormal vs. normal)	1.902	1.034, 3.50	0.039*	10.94	2.92, 41.02	< 0.001*

*HR* hazard ratio, *CI* confidence interval, *HCV* hepatitis C virus, *β2m* beta-2-microglobulin, *ISS* International Staging System, *ALT* alanine transaminase, *AST* aspartate aminotransferase, *T.bil* total bilirubin

*Significant at *p* ≤ 0.05

**Table 5:** Kaplan-Meier analysis for DFS between the different ISS stages

ISS stages	Mean DFS	95% CI	Median	95% CI	***P*** value
I	22.905	0.0, 46.6	5.000	0.0, 10.4	0.587
II	30.537	14.6, 46.5	16.000	11, 21	
III	24.933	15.4, 34.5	14.000	3.9, 24.1	

*Significant at *p* ≤ 0.05

**Table 6:** Kaplan-Meier analysis for DFS between the different treatment regimens

	Mean DFS	95% CI	Median	95% CI	***p*** value
Endoxan-Dexa	4.444	0.6, 8.3	3	0.0, 11.8	< 0.001*
VCD	29.511	16.4, 42.6	14	6.8, 21.2	
VRD	25.613	16.1, 35.2	24	17.5, 30.5	
VTD	44.063	24, 64.1	-	-	

*Significant at *p* ≤ 0.05

*VCD* Velcade® (bortezomib)-cyclophosphamide-dexamethasone, *VRD* Velcade® (bortezomib)-Revlimid® (lenalidomide)-dexamethasone, *VTD* Velcade® (bortezomib)-thalidomide-dexamethasone, *Endoxan-Dexa* endoxan-dexamethasone

## Discussion

Multiple myeloma is a B cell malignancy involving terminally differentiated, non-dividing plasma cells that survive for months to years in the bone marrow and produce antigen-specific immunoglobulin, thus forming an integral part of the immune defense system [15]. For MM patients, the impact of preexisting HCV infection is unclear, and also, the mechanisms by which HCV may cause myeloma are less explored. The aim of the current study is to explore the clinical characteristics and prevalence of the hepatitis C virus infections in Egyptian multiple myeloma patients. In our study, the prevalence of HCV infections in Egyptian myeloma patients revealed that 15/81 (18.5%) patients were anti-HCV positive. For more accurate confirmation, RT-PCR was done. Out of 15 anti-HCV positive patients, 9 (11.1%) patients are HCV RNA negative while the remaining patients (6/15; 7.4%) are HCV RNA positive. Several studies have explored the prevalence and pathogenic role of HCV infection in hematological malignancies especially in lymphoproliferative disorders (LPDs), including MM. There is heterogeneity in identifying HCV infection status, with the majority of studies defined HCV infection as positivity for HCV antibody only such as Cavanna et al., Musto et al., Teng et al., and Takeshita et al. who reported a higher prevalence of HCV infection in LPDs patients including MM (16.7%, 11.1%, 9%, and 4.9%), respectively [13, 16–18]. However, Silvestri et al. revealed that the prevalence and relative risk of being infected by HCV is higher in B non-Hodgkin lymphoma but not MM patients [19]. Moreover, Vener et al. and Anderson et al. reported no increase of HCV prevalence in patients with MM (0.7% and 0.2%), respectively [20, 21]. Okan et al. defined HCV positivity as positivity for both HCV antibody and HCV RNA, and this study revealed no significant difference in the combined prevalence of HBV and HCV infections in patients with LPD [22], whereas De Rosa et al. defined HCV positivity as positivity in either HCV antibody or HCV RNA and reported 16.1% prevalence of HCV infection in patients with B-LPD including MM [23]. The incidence of hepatic adverse events in our MM patients revealed that hyperbilirubinemia with grade 4 adverse events was significantly higher in the anti-HCV positive/HCV RNA positive group versus anti-HCV negative group (16.7% vs. 1.5%, *p* value = 0.005). This is in accordance with Teng et al. who found that MM patients who were chronic viral hepatitis carriers experienced more and earlier hepatic adverse events during treatment which could not be explained by the difference in the therapies received [13]. Finally, as MM is a B cell lymphoproliferative disease, the possible HCV carcinogenic effect leading to lymphoma development may also play a role in MM development. HCV directly changes the lymphocyte phenotype through different types of protein, indirectly fuel the growth of B cells or an unknown infectious agent may affect lymphomagenicity in HCV infection [24]. Moreover, HCV viral proteins have been identified in B cells of HCV-infected patients. In addition, there was upregulated B cell receptor (BCR) signaling in human primary B cells of HCV-infected patients [25], which may contribute to a better understanding of the molecular mechanisms underlying MM. In addition to the above mechanisms by which HCV may cause myeloma, there is a therapeutic dilemma in the clinical settings for treating MM patients infected by HCV. Several studies found HCV reactivation or acute exacerbation in HCV-infected patients taken chemotherapeutic agents, such as protocols including corticosteroids or thalidomide [26]. Corticosteroids may enhance HCV viral RNA replication or suppress HCV-mediated immune response [27]. Also, thalidomide may inhibit activation of IκB kinase and signaling of nuclear factor-κB, which leads to enhanced replication of HCV viral RNA [28]. Notably, Mahale et al. [29] revealed that chemotherapeutic agents may not lead to viral relapse in cancer patients infected with HCV and treated with antiviral drugs. Therefore, early HCV infection identification and treatment is necessary before starting chemotherapeutic agents in MM patients. Our data showed that patients who receive VTD as induction therapy have higher DFS. Though there is a selection bias in treatment considerations for our patients, as patients with younger age and higher risk or higher disease burden (by β_2_M or PET scan), were considered to receive VRD. So this explains the poorer survival in this group. Patients living at distant areas from our department, who have difficulties in coming to the clinic weekly, receive VTD as induction, while VCD remains the most common standard regimen given to our patients and this explains the moderate survival for this group. Finally, limitations of this study include small sample size; thus, this work should be extended on wider prospective clinical studies with larger patient series and longer follow-up period to assess the clinical impact of HCV infection in Egyptian MM patients and to validate whether increased risk of MM in patients with HCV infection is due to its actual risk factor or due to high prevalence of HCV infection in our population. In addition, the association between cytogenetic abnormalities in MM patients and HCV infection warrants further study to clarify the relationship.

## Conclusion

In conclusion, our study revealed the prevalence of HCV infection in Egyptian MM patients. Serologic tests at diagnosis are necessary to identify these patients, and confirmation of positive cases by molecular techniques should be mandatory, with regular follow-up for liver dysfunction. Finally, further larger studies explaining the molecular mechanisms linking HCV and the MM pathogenesis are warranted.

## Data Availability

The datasets used and/or analyzed during the current study are available from the corresponding author on reasonable request.

## Abbreviations

ALT: Alanine transaminase
ASCT: Autologous stem cell transplantation
AST: Aspartate aminotransferase
BCR: B cell receptor
BM: Bone marrow
CI: Confidence interval
CMIA: Chemiluminescent microparticle immunoassay
CR: Complete remission
DFS: Disease-free survival
Endoxan-Dexa: Endoxan-dexamethasone
FLC: Free light chain
HCC: Hepatocellular carcinoma
HCV Ab: Hepatitis C virus antibodies
HCV: Hepatitis C virus
HR: Hazard ratio
ISS: International Staging System
LPDs: Lymphoproliferative disorders
MGUS: Monoclonal gammopathy of undetermined significance
MM: Multiple myeloma
OS: Overall survival
PCR: Polymerase chain reactions
PCs: Plasma cells
PD: Progressive disease
POEMS: Polyneuropathy, organomegaly, endocrinopathy, monoclonal gammopathy, and skin changes syndrome
PR: Partial response
SD: Standard deviation
ST: Stationary disease
SVR: Sustained viral response
T.bil: Total bilirubin
VCD: Velcade® (bortezomib)-cyclophosphamide-dexamethasone
VGPR: Very good partial response
VRD: Velcade® (bortezomib)-Revlimid® (lenalidomide)-dexamethasone
VTD: Velcade® (bortezomib)-thalidomide-dexamethasone
β_2_m: Beta-2-microglobulin
